# Hypofractionation and Stereotactic Body Radiation Therapy in Inoperable Locally Advanced Non-small Cell Lung Cancer

**Published:** 2021-04-22

**Authors:** Mikel Rico, Maribel Martínez, Maitane Rodríguez, Lombardo Rosas, Andrea Barco, Enrique Martínez

**Affiliations:** ^1^Department of Radiation Oncology, Complejo Hospitalario de Navarra, Pamplona 31008, Navarra, Spain; ^2^Health Research Institute of Navarre (IdiSNA), Navarra Biomed, Pamplona 31008, Navarra, Spain

**Keywords:** non-small-cell lung cancer, hypofractionation, stereotactic body radiation therapy, radiotherapy

## Abstract

**Background and Aim:**

Radiotherapy (RT) plays a key role in the control of locally advanced non-small cell lung cancer (LA-NSCLC). Throughout the years, different doses and fractionations of RT have been used in an attempt to optimize the results. Recently, special interest has been given to hypofractionation (hypoRT) and stereotactic body radiation therapy (SBRT). HypoRT is a relatively widespread treatment, although the accompanying level of evidence is limited. For its part, SBRT has been used specially to overdose specific areas of the disease as a boost after radiochemotherapy. In both cases, the study of how to integrate these RT tools with chemotherapy and immunotherapy is fundamental. In addition, the 2020 COVID-19 pandemic situation has sparked increased interest in hypofractionated treatments. In this review, we analyze the role of SBRT and hypoRT in the management of LA-NSCLC in accordance with current scientific evidence.

**Relevance for Patients:**

The objective of this article is to introduce professionals to the role that hypoRT and SBRT can play in the treatment of LA-NSCLC to offer the best treatment to their patients.

## 1. Introduction

The Pacific study showed how the administration of durvalumab after radiotherapy (RT) and chemotherapy (CRT) significantly improved survival in patients with unresectable Stage III non-small cell lung carcinoma (NSCLC). It was an important milestone in the treatment of this disease [1]. Studies have shown that the most common type of recurrence in LA-NSCLC is distant [2,3]. Despite this, locoregional control is still essential to increase survival, so it is of great interest to optimize RT treatments [4]. Over the years, different strategies have been used to improve the results with RT by altering the doses, the fractionations and applying the latest technological advances. In recent times, special emphasis has been placed on the use of hypofractionated treatments. The progressive knowledge and expansion of stereotactic body RT (SBRT) has prompted the study of its application in this disease. In addition, with the impact of the COVID-19 pandemic, different scientific societies gave recommendations aimed at shortening treatment times by paying special attention to hypofractionated treatments. Finally, the emergence of immunotherapy has meant a true paradigm shift in the management of lung cancer. The best way to combine RT with these new treatments remains to be defined. In this context, in this publication, we intend to review the status of hypoRT and SBRT in the changing scenario of locally advanced and unresectable lung cancer.

## 2. Materials and Methods

We searched the National Comprehensive Cancer Network, the European Society for Medical Oncology (ESMO), European Society for Radiation Oncology (ESTRO), and the American Society for Radiation Oncology updated guidelines. The following search strategy was performed of the PubMed database on July 2020: (lung AND (non-small cell OR NSCLC) NOT metast * [TI]) AND (stage III OR locally advanced OR locally-advanced) AND (radiation therapy OR radio -therapy) AND (hypofract * OR hyperfract * OR adaptive RT OR SBRT) NOT case reports. Clinical studies, clinical trials, meta-analysis, and reviews were selected. References were also analyzed. We classified in hyperfractionation (hyperRT), hypofractionation (hypoRT), adapted therapy, or SBRT.

## 3. Results and Discussion

### 3.1. State of the art: Current management, conventional dose escalation, and hyperfractionation

Treatment of Stage III N0 or N1 NSCLC consists of surgery followed by adjuvant treatment. When there is mediastinal involvement, the role of surgery is controversial. A 2007 Phase III study of the European Organization for Research and Treatment of Cancer (EORTC) randomized CT followed by RT or surgery to N2 patients and showed no differences between both treatments [5]. Regarding neoadjuvant treatment, there is evidence of the benefit of CT as neoadjuvant treatment in Stage III [6]. Considering neoadjuvant CRT, a Phase III intergroup study randomized to CRT followed by surgery versus exclusive CRT. Although no differences in survival were demonstrated between the two groups, an unplanned analysis found that those patients treated with lobectomy had a longer survival when compared to patients treated with CRT [7]. In any case, the role of surgery in N2 patients is still subject to debate in part due to the great diversity of this group of patients. There is evidence to suggest that the prognosis after surgery depends on the burden of mediastinal involvement [8]. In this way, in cases with few affected lymph nodes or in a single station, surgery could be indicated.

In unresectable situations, the evidence favors treatment with RT at doses of 60 Gy–70 Gy in 2 Gy fractions concomitant with CT and followed by durvalumab, based on the results of the Pacific trial [1,9,10]. In this study, patients received concurrent CRT and the administration or not of adjuvant durvalumab was randomized. Latest data reported at ESMO 2020 maintained impressive results with increased follow-up. In the experimental arm with durvalumab, median overall survival (OS) was 47.5 months and 4-year OS was 49.6%, while in the placebo arm, the median survival and 4-year OS were 29.1 months and 36.3%, respectively [10]. Anyway, in Europe, durvalumab is only approved by the European Agency of Medicaments (EMA) for patients with PDL1> 1% [11].

In non-randomized studies, RT dose escalation has shown improvements in survival and local control (LC) both in exclusive RT and administered with CT [12-16]. OS in these studies ranged from 19 to 24 months. In an analysis by Machtay *et al.*, 1356 patients drawn from Radiation Therapy Oncology Group (RTOG) clinical trials were retrospectively analyzed. This study showed that 1% increases in bioequivalent dose (BED) were related with 3% improvement in LC and 4% relative improvement in OS [17].

Based on these encouraging results, the Phase III trial RTOG 0617 trial explored the benefits of increasing the dose of RT by randomizing patients to receive 74 Gy versus 60 Gy. This trial was prematurely closed due to the low survival in the experimental arm. Median OS in the control arm was 28.7 months (the same as in the control arm in the Pacific study) and the 2-year OS was 58%, while in the experimental arm, median OS and 2-year OS were 20.3 months and 45%, respectively. Surprisingly, the LC and locoregional control were also worse in the experimental arm. It was concluded that increasing the dose to 74 Gy provided no benefits [18]. There were several coincidence factors in the experimental arm that may have contributed to these results, such as the increased doses received by the heart, less adherence to RT protocols, worse compliance to the CT schemes, and increased toxicity deaths. Finally, the use of intensity modulated RT (IMRT) and the volume of patients treated by the participating centers may have influenced the results [19-22].

Although the main clinical guidelines propose conventional fractionation RT as the standard of care [9,23,24], the controversy about the benefit of dose escalation remains open. In 2016, Ramroth *et al*. published a meta-analysis examining studies with patients randomized to different RT schemes, including regimes with splits, hypoRT, hyperRT, or dose escalation with conventional fractionation. The results showed that the increased of BED administered without CT improved survival [25].

On the other hand, it is known that the prolongation of the global treatment time has a negative impact on the control of the disease as a consequence of the accelerated repopulation of cells, which could also contribute to the results of the RTOG 0617 trial. For each day that RT treatment is prolonged beyond 6 weeks, the chances of survival are reduced by 1.6% [20,21,26,29]. Based on this, other ways to elevate BED without prolonging the overall treatment time have been explored.

### 3.2. Hyperfractionation

HyperRT consists in increasing the total number of fractions delivered twice (or more) per day, at a reduced dose per fraction and it has shown uneven results.

Treatment with continuous hyperfractionated accelerated RT (CHART), administering 54 Gy in 12 days, in three 1.5 Gy fractions per day, showed an increase in survival [30]. However, the same scheme without treatments on the weekends (CHARTWEL) showed no benefit over conventional treatment [31]. Similarly, an Australian study on hyperRT and the study RTOG 9410 comparing various treatment schemes including hyperRT did not show benefit through this strategy [32,33]. A meta-analysis analyzed 2000 patients from 10 trials comparing conventional fractionation with hyperRT showed a survival benefit with hyperRT of 3.8% and 2.5% at 3 and 5 years, respectively, but with a significant increase in acute esophagitis (9% vs. 19%) [34].

The diffusion and widespread implementation of hyperRT has been hampered by logistical barriers. Furthermore, it is not well known how these treatments should be supplemented with CT. Finally, hyperRT has been consistently observed to increase esophageal toxicity. Therefore, although the 2019 National Institute for Health and Care Excellence (NICE) guidelines propose hyperRT as an alternative to conventional fractionation in patients not candidates for CRT treatment, its use is minority [35,36].

### 3.3. Hypofractionation

Nowadays, there is a greater interest in exploring fractionations aimed at shortening the global treatment time by increasing the doses per fraction and thus increasing the BED. It has been observed that there is a moderate linear relationship between BED and OS when using hypoRT so that for each 1 Gy increase in BED, there would be a benefit of 0.36–0.7% in OS [37]. In most of the published studies, they use moderate hypoRT with doses of 2.5–3 Gy per fraction.

#### 3.3.1. Hypofractionation without concurrent CT

It is important to highlight that patients in the Pacific and RTOG 0617 trials had a performance status (PS) of 0 or 1 and median age of 64 years, what, undoubtedly, has its implications in the results. In routine clinical practice, patients’ PS is frequently ≥2, median age at diagnosis is 70 years, and they often have comorbidities that jeopardize treatment strategy. In fact, it is estimated that between 55% and 59% of the patients are not candidates for concurrent CRT [38,39]. Some experiences have shown good results with acceptable toxicity using hypoRT in this group of patients [40]. A retrospective Spanish study analyzed the results treating patients no candidates to CRT with hypoRT (66 Gy in 24 fractions). In Stage III subgroup, 2-year OS was 37.5% and no Grade 3 toxicity was reported [41]. Din *et al*. published a retrospective analysis with 609 patients treated with hypoRT (55 Gy in 20 fractions of 2.75 Gy) without concurrent CT (28% received sequential CT). Median OS and 2-year OS for locally advanced disease were 20 months and 40%, respectively, with no Grade 3 or 4 toxicities [42]. Another retrospective analysis by Amini *et al.*, with 300 patients, compared hypoRT (45 Gy in 15 fractions of 3 Gy) with conventional fractionations in patients not candidates for CRT treatments. They concluded that hypoRT was an acceptable treatment option for poor PS patients, with similar results to those achieved with conventional RT [43].

Recently, it has been published an analysis of the National Cancer Database (NCDB) comparing hypoRT versus conventionally fractionated RT in patients treated with RT alone. A total of 6490 patients were evaluated, 5378 with conventional RT (median dose of 66 Gy in 2 Gy) and 1112 with hypoRT (median dose 58.5 Gy in 2.5 Gy fractions). HypoRT was associated with older age, lower BED, academic facility type, higher T-stage, and lower N-stage. After adjusting by these covariates, no difference in OS was observed between both groups [44].

In a review by Kaster *et al.*, analyzing the studies with hypoRT without concurrent CT, the reported weighted mean acute esophageal and pulmonary toxicity were 1.9% and 1.2%, respectively. Late esophageal and pulmonary toxicity were 1.4% and 6.9%, respectively. Two-year survival ranged from 18% to 42%. Toxicities were defined as events that could be scored as Grade 3 or more [37].

In a 2019 review, Parisi *et al*. analyzed up to 29 studies published since 2007. In hypoRT treatments without CT, the dose range ranged from 45 to 85.5 Gy. Acute grade ≥3 esophageal toxicity was 0–15% and acute pulmonary toxicity was 0–44%. The late esophageal and pulmonary toxicity found was 0–16% and 0–47%, respectively, with pulmonary toxicity being most commonly ≤Grade 3. Two-year OS ranged from 22 to 68.7% [45].

More aggressive hypoRT schemes have been employed, although, as with SBRT, the central location of the lesions is of particular concern. In a Phase I dose escalation trial, 55 patients with poor PS were treated with doses of 50, 55, or 60 Gy in 15 fractions. It was concluded that precision hypoRT with 60 Gy in 15 fractions is generally well tolerated [46]. The same group developed a Phase III trial in which patients with a PS ≥2, not candidates for concurrent CRT, were randomly assigned to either 60 Gy in 30 fractions or 60 Gy in 15 fractions. In an interim analysis, the median OS was 11.5 months with no intergroup differences. The authors concluded that hypoRT may be an alternative for these groups of patients [47]. A retrospective analysis published in 2020 with 42 patients with KI ≥70% treated with doses of 60 Gy in 15 fractions (and mostly with sequential CT), showed a 2-year survival of 69%, with 14% esophageal toxicity ≥G3 and 14% pulmonary toxicity ≥G3 [48].

With the emergence of immunotherapy in the treatment of LA-NSCLC, the use of hypoRT treatments is controversial. On the one hand, there is a concern of a cumulative risk for severe pneumonitis. On the other hand, the immunomodulatory role of hypoRT could increase the effectiveness of treatments. In this sense, the Phase II TRADE-hypo trial will investigate two radiation regimens combining durvalumab therapy with either conventionally fractionated or hypoRT (55 Gy in 20 fractions), in patients not candidates for CT. Another Phase II study (DUART trial) will explore the role of durvalumab after RT in patients not candidates for CT, also including hypofractionated schemes. Both studies are in recruitment [49,50].

During the COVID-19 pandemic, many recommendations have emerged by different societies with the common message that hypofractionated schedules without concurrent CRT are appropriate [51-53]. In the practical guideline carried out by ESTRO, there is consensus to recommend hypoRT alone or with sequential CT. The most recommended regimens are 60 Gy in 20 fractions, 60–66 Gy in 24–30 fractions, and 55 Gy in 20 fractions [52].

As we can see, there is great heterogeneity in the doses and fractionation schemes published in the literature. In any case, and despite the difficulty in obtaining conclusions about the efficacy of hypoRT in patients not candidates for concurrent CRT, these treatments are increasingly becoming part of routine clinical practice.

#### 3.3.2 Hypofractionation with concurrent CRT

The Phase I trial called Alliance, studied RT dose escalation using advanced RT techniques with weekly carboplatin-based concurrent CT. The daily fractionation was escalated from 2.22 Gy to a maximum of 3 Gy per fraction to a total fixed dose of 60 Gy over four planned cohorts. The MTD was reached and defined as 60 Gy in 24 fractions of 2.5 Gy [45]. In other small Phase I study with 3DRT, 13 patients were treated with 3 Gy per fraction with concurrent CT. The MTD was 69 Gy at 3 Gy per fraction with no treatment related deaths reported [55].

In the systemic review by Kaster *et al.*, in the hypoRT with concurrent CT subgroup, the weighted mean acute esophageal and pulmonary toxicity were 14.9% and 7.9%, respectively. Weighted mean late esophageal and pulmonary toxicity were 16.6% and 12.2%, respectively. The 2-year OS ranged from 24% to 58% [37]. In the previously referenced review by Parisi *et al.*, in the hypoRT treatments with concurrent CT, the acute Grades 2 and 3 esophagitis ranged between 3% and 41.7% and acute pneumonitis ranged from 0 to 23%. Late esophageal and pulmonary toxicity ranged from 0 to 8.3% and from 0 to 47%, respectively. The 2-year survival was 38.6–68.7% [45]. These results reflect, with the limitations inherent in this type of study, an increase in toxicity in combined hypoRT-CT treatments.

In 2014, the SOCCAR trial compared concurrent versus sequential hypoRT (55 Gy in 20 fractions of 2.75 Gy) and CT. Initially, it was planned to be a Phase III study, but due to poor recruitment, it was restructured to a Phase II study. The main objective was to assess the tolerability of the treatment. The results showed low toxicity, with 9.3% and 8.2% Grade 3 esophageal toxicity in the concurrent and sequential arms, respectively. Two-year OS in the concurrent CRT arm was 50% versus 46% in the sequential arm. The median OS for concurrent versus sequential was 24.3 and 18.4 months, respectively.

In 2019, a retrospective analysis was published with 100 patients treated with hypoRT (55 Gy in 20 fractions) with 2 cycles of concurrent CT followed by 2 cycles of adjuvant CT with vinorelbine and cisplatin. The 2-year OS was 58%, higher than in the SOCCAR study, possibly due to the incorporation of advances in disease staging and modern RT techniques [56]. Although this scheme of RT with concurrent CRT has not ever been directly compared with conventional CRT, it has not hindered it from being the most employed RT fractionation in the UK, according to a survey carried out about the most common practices in the treatment of NSCLC [36]. In fact, in the NICE guidelines for NSCLC updated in 2019, this scheme is presented as an alternative for radical treatments [35].

The EORTC 08972-22973 study randomized hypoRT (66 Gy in 24 fractions of 2.75 Gy) with concurrent CT (daily low-dose cisplatin) versus sequential CT (two cycles of cisplatin and gemcitabine). The study was prematurely discontinued due to poor recruitment, and no significant differences were seen between the two arms, maybe because of the poor power of the study. The median OS and 2-year OS for the concurrent arm were 16.5 months and 39%, respectively. Acute G3 esophagitis was higher in concurrent than sequential CRT (14% vs. 5%). Anyway, this 2007 study, employed elective nodal irradiation and old planning techniques [57,58].

This 66 Gy in 24 fractions scheme is still used in common clinical practice in some centers incorporating new planning and delivery techniques and positron emission tomography (PET)-based nodal treatment. In a Phase II trial, the addition or not of cetuximab was randomized to concurrent CRT (66 Gy in 24 fractions and low-dose cisplatin). The results were excellent independently of the administration of cetuximab, with a median OS of 31.5 months and 2-year OS of 59.4%. On the other hand, it was observed that the dose on the esophagus, the PS, and the comorbidities of the patients influenced the OS [50]. A retrospective analysis with 469 patients was published in 2017 using the same scheme of hypoRT and low-dose CT. The authors found a significant association between heart dose and OS [60]. This shows the importance of selecting cases for hypoRT treatments with concomitant CT, as well as optimizing RT techniques and adjusting CT treatments. The use of 4DCT, IMRT, volumetric modulated arc therapy (VMAT), and advanced image-guided RT (IGRT) techniques such as cone-beam CT (CBCT) may be especially necessary when performing this type of treatment to try to minimize esophageal, cardiac, and pulmonary toxicity.

The ESTRO recommendations for the COVID-19 pandemic argue against the use of hypoRT with concurrent CT [61]. However, the British group proposes the use of hypoRT with concurrent CT as per SOCCAR protocol for selected cases [53]. This divergence reflects the limitations of the current evidence to be able to give firm recommendations in this regard. Going further, a recent systematic review has questioned the benefit of performing concurrent CRT treatments over sequential treatment when the dose is increased through fractionation modifications [62].

### 3.4. Personalized hypofractionated radiation therapy

Typically, RT treatments use fixed doses of radiation for a particular disease. However, the possibility of individualizing dose escalation based on the tolerance limits of healthy organs has been explored. This has been called isotoxic radiation therapy [63]**.**

Cannon *et al*. published a Phase I dose escalation study using isotoxic hypoRT without concurrent CT. In this study, patients were treated in 25 fractions, escalating the dose from 2.28 Gy to 3.42 Gy individually, according to the risk of developing pneumonitis. The MTD was defined as 63.25 Gy in 25 fractions of 2.53 Gy, similar to other dose escalation studies. Late Grade 4–5 toxicities were attributable to damage to central and perihilar structures and were correlated with dose to the proximal bronchial tree [64]. Once again, we verify the importance of limiting the dose received by the central structures. The importance of employing advanced RT techniques is evident if the dose is to be increased through hypofractionation.

The IDEAL-CRT trial evaluated dose escalation up to 73 Gy in 30 fractions over 6 weeks with dose escalation calculated on an individual patient basis according to either lung or esophageal radiation dose. Median OS was 37.5 months and 2-year OS was 62.9% [65,66].

The ADSCAN Phase II study, currently in recruitment, includes different forms of fractionation for patients treated with CT and sequential RT. It randomizes to five different RT schemes: STANDARD RT: 55 Gy in 20 fractions; CHART-ED: 54 Gy (three fractions of 1.5 Gy/day for 12 days), followed by 10.8 Gy (two fractions of 1.8 Gy/day for 3 days); IDEAL: 30 daily fractions of isotoxic RT (63–71 Gy); I-START: 20 daily fractions of isotoxic RT (55–65 Gy); and ISOTOXIC-IMRT: bidaily fractions of isotoxic RT over 4 weeks (max dose 79.2 Gy). The aim of this study is to find the most promising way to increase doses to subsequently develop a Phase III trial [67].

Other studies have used PET to adapt the volume of treatment. In a Phase II carried out by Kong *et al.*, a PET was performed at 40–50 Gy to redefine the treatment target and a hypoRT boost was administered on the observed residual disease. The threshold dose was defined as the dose over which the risk of Grade 2 pneumonitis was above 17.2% (approximately equivalent to 20 Gy mean lung dose). In this study, the median dose administered over the tumor was 83 Gy in 30 fractions and most patients received CT concurrently. LC at 2 years was 82% and median OS was 25 months [68].

The randomized Phase II PET-boost trial aimed to improve LC by boosting either the whole primary tumor (arm A) or the high FDG uptake region inside the primary tumor (arm B). The boost dose was maximized by normal tissue constraints (isotoxic treatment). The results were presented at ESTRO 2020 congress, showing a median total dose of 78 Gy for the arm A and 84 Gy for the arm B and a LC >90% at 1 year for both arms, respectively, although the trial did not reach predefined sample size and many scans were not evaluable [69].

The RTOG 1106 is a Phase II study with a control arm (60 Gy in 30 fractions) and an experimental arm (21 fractions at 2.2 Gy and 9 fractions applied on the residual disease seen in a PET, at doses between 2.2 and 3.8 Gy without exceeding the mean lung dose of 20 Gy). The experimental arm is a hypofractionated, adaptive, and isotoxic scheme [70]. The first results of this study were presented at World Conference on Lung Cancer 2020. Adaptive RT increased numerically local and local-regional control, but these differences were not statistically significant. There were no differences in Grade 3 or worse toxicity, OS, progression-free survival, and lung cancer-specific survival between treatment arms [71].

Although this type of treatment can make it possible to increase the dose on the disease, there is still no clear evidence about its clinical benefit. In the future, studies exploring this type of fractionation should include immunotherapy and consider customizing the RT dose based on radiosensitivity profiles [72].

### 3.5. SBRT

Conventionally, wide margins have been used to compensate movement of thoracic lesions during the respiratory cycle, thus limiting the radiation dose that could be delivered. SBRT consists of the administration of high doses of radiation with high precision with a narrow margin and with a strong gradient to protect the surrounding healthy tissue. To be able to carry this out, it is essential to have 4DCT images, use strategies to compensate for the movement during the respiratory cycle (as dampening, gating, active breathing control, or tracking) and to have a good IGRT system during treatment. As IGRT strategy, the use of CBCT and more recently 4D-CBCT are widely disseminated [56,57]. In this way, it has become feasible to administer radiation doses hitherto unimaginable with limited toxicity [58]. It is of interest to note that some of the strategies used in SBRT to improve precision can be similarly used in hypoRT treatments to reduce possible toxicity, as previously noted.

SBRT achieves LC in more than 90% of patients with Stage I NSCLC and increases survival when compared to conventional RT, becoming the alternative to surgery in inoperable patients [73,74]. From this experience, the SBRT role at LA-NSCLC has become an area of great interest. The objective of SBRT in this context would be to optimize LC of the disease while trying to minimize toxicity. However, to this day, it is still unclear how best to integrate SBRT with established treatments.

There are several publications that have proposed different treatment schemes, paying special attention to its safety. The University of Kentucky group published a tolerability study in which the patients were treated with CRT (60 Gy in 30 fractions) and then SBRT was administered on the residual tumor (<5 cm) observed in PET. The SBRT dose was 20 Gy in two fractions and 19.5 Gy in three fractions for central lesions, always above 100 Gy BED (alpha/beta = 10). Thirty-seven patients were treated with a follow-up of 25.5 months. Median OS was 25.2 months LC was 78%. Grade 3 pneumonitis occurred in 13.5%. Two patients died due to fatal hemorrhage although no dosimetric differences were seen. Anyway, they proposed 175 Gy (BED10) as an estimated MTD for combined CRT and SBRT boost planning for the pulmonary artery for future studies. The authors concluded that it is a safe treatment resulting in good LC with not increased risk for toxicity above that for standard radiation therapy [75,76].

Other groups have developed Phase I dose escalation studies with SBRT toxicity boost following CRT, but with small number of patients and limited follow-up. Hepel *et al*. carried out a Phase I dose escalation study after CRT with 50.4 Gy, exploring four dose levels: 16 Gy in two fractions, 20 Gy in two fractions, 24 Gy in two fractions, and 28 Gy in two fractions. One-year locoregional control was 100% with boost doses ≥24 Gy. One patient died of bronchopulmonary hemorrhage associated to the dose applied to the proximal bronchovascular tree. Based on their results, the authors recommend limiting the doses applied to the bronchovascular tree or increase the number of fractions [77]. In a previous dosimetric pilot study, the authors proposed dose limits for the organs at risk and showed how it is feasible to respect these constraints, but there were no predetermined limits for the bronchovascular tree [78]. Higgins *et al*. analyzed four dose levels (18 Gy in two fractions, 20 Gy in two fractions, 30 Gy in five fractions, and 35 Gy in five fractions) following concurrent CRT at 44 Gy. Two patients developed Grade 5 toxicities (a tracheoesophageal fistula and one case of hemoptysis). The authors concluded that although 30 Gy in five fractions is the maximum tolerated dose calculated, 20 Gy in two fractions may be a reasonable dose as no Grade 5 toxicities were observed with this scheme [79]. In a retrospective study, 16 patients received conventional CRT to a median dose of 50.40 Gy followed by an SBRT boost with an average dose of 25 Gy given over five fractions. One-year LC was 76%, 25% developed Grade 2 acute pneumonitis, and no Grade 5 toxicities were observed [80]. In Table 1, we collect and summarize these studies.

**Table 1:** Stereotactic body radiotherapy as boost after chemoradiotherapy

Publications	N	RT dose in CRT	SBRT dose	Cumulative BED10	Local control	Survival	Toxicity
Kumar *et al*. [75]	37	60 Gy	10 Gy×2fx 6.5 Gy×3fx for central tumors	110 Gy 102.2 Gy	78%	25.2 m OS	13.5% Grade3 5.4% Grade 5 (fatal hemorrhage)
Hepel *et al*. [77]	12	50.4 Gy	8 Gy×2fx 10 Gy×2fx 12 Gy×2 fx 14 Gy×2fx	88.3 Gy 99.5 Gy 112.3 Gy 126.7 Gy	1 year – LRC: 78% 1 year – LRC<24 Gy: 50%, 1 year – LRC≥24 Gy: 100%	1 year – OS: 67%	8% Grade 3–5 (fatal hemorrhage)
Higgins *et al*. [79]	19	44 Gy	9 Gy×2fx 10 Gy×2fx 6 Gy×5fx 7 Gy×5fx	87 Gy 92.8 Gy 100.8 Gy 112.3 Gy	3 years – LRC: 56%	3 years – OS: 39%	10.5% Grade 5; (tracheoesophageal fistula and fatal hemoptysis) With 10 Gy×2fx: no grade≥3
Karam *et al*. [80]	16	50.4 Gy (45 Gy-60 Gy)	4–6 Gy×5 fx	97 Gy (81 Gy–120 Gy)	1 year – LC: 76%	1 year – OS: 78%	25% Grade 2 acute pneumonitis. No grade≥3

N: Number of patients; RT: Radiotherapy; CRT Chemoradiotherapy; SBRT: Stereotactic body radiotherapy; fx: Fractions; LC: Local control; LRC: Locoregional control; OS: Overall survival; m: Months; n.a.: Not available.

In a study performed by a Korean group in 2018, Kim *et al*. combined CRT for nodal areas and SBRT on the primary lesion, when the targets were distant from each other. With 21 treated patients, 2-year OS was 60.5%, there were no relapses regarding the primary tumor and 14% of the cases developed Grade 3 pneumonitis, all in patients aged over 79 years. The methodological limitations of the study do not allow drawing conclusions, but it opens the door to another possible therapeutic application for SBRT. The use of SBRT has also been examined as part of a multimodal treatment with CRT and surgery in the locally advanced disease. The currently on-going Linnearre I is a Phase I viability study in which SBRT is given as a neoadjuvant treatment in N0-N1 patients [81]. In other prospective study published in 2018 by Singh *et al.*, SBRT was employed as an adjuvant treatment after surgery and before adjuvant CRT. A 10 Gy single fraction was applied on the affected nodal stations or in cases of positive margins with good results in terms of LC [82].

The Phase I hybrid study proposes combining CT with hypoRT on lymph node disease (24 fractions of 2.42 Gy) with SBRT (3 fractions of 18 Gy) on the primary tumor, which must be peripheral and smaller than 5 cm. This way, the aim is to increase the dose on the primary tumor, without increasing the dose at centrally to avoid major toxicity. This study has completed recruitment [83].

Following the publication of the Pacific study, interest has grown about how to combine SBRT with CRT and durvalumab treatment. The immunomodulatory role of SBRT makes it a particularly interesting tool in this context [84].

It is known that RT can induce an immune response that acts against the tumor by increasing immunogenic cell death and stimulating a systemic immunity against the disease. However, this effect seems to be counteracted by the immunosuppressive capacity of the tumor microenvironment itself. The synergistic role that can be established between RT and drugs that reduce the immunosuppressive capacity of the tumor is currently under investigation. The effect that dose and fractionation may have on the immunomodulatory capacity of RT is also being investigated, although there is evidence that the immunogenic response is greater when high doses per fraction are used [85]. For this reason, it is of the utmost interest to try to combine immunotherapy with SBRT, which may contribute to improving LC of the lesion both due to its direct role against the tumor and through its immunoregulatory contribution. In this context, a Phase II study consisting of the administration of CRT followed by durvalumab and a boost of 20 Gy in 2–3 fractions on the primary tumor has started recruitment [86]. This type of schema is a model of how LA-NSCLC might be handled in the future.

## 4. Conclusions

HypoRT can shorten the treatment time, which may provide clinical benefit by reducing the repopulation of tumor cells during RT. In addition, it allows a more efficient use of services. The efficacy of hypoRT regimens with or without CT should be contrasted in prospective studies designed for it. These studies should introduce technological advances in the field of RT as well as immunotherapy, as they are already part of the LA-NSCLC treatment. For its part, SBRT treatments may increase LC in patients with LA-NSCLC with an acceptable safety profile, although the level of evidence is still poor.

Given the immunomodulatory role of RT and especially of SBRT, it is presumable that, in the coming years, new treatment schemes will be proposed that tend to integrate hypoRT and boost with high doses of RT per fraction together with immunotherapy. Undoubtedly, it is a research area full of possibilities that in the future will bring great changes in the management of the LA-NSCLC.
